# CERENKOV2: improved detection of functional noncoding SNPs using data-space geometric features

**DOI:** 10.1186/s12859-019-2637-4

**Published:** 2019-02-06

**Authors:** Yao Yao, Zheng Liu, Qi Wei, Stephen A. Ramsey

**Affiliations:** 1 School of Electrical Engineering and Computer Science, Oregon State University, Corvallis, 97330 OR USA; 2 Department of Biomedical Sciences, Oregon State University, 106 Dryden Hall, Corvallis, 97330 OR USA

**Keywords:** SNP, GWAS, noncoding, rSNP, Data space, Machine learning

## Abstract

**Background:**

We previously reported on CERENKOV, an approach for identifying regulatory single nucleotide polymorphisms (rSNPs) that is based on 246 annotation features. CERENKOV uses the xgboost classifier and is designed to be used to find causal noncoding SNPs in loci identified by genome-wide association studies (GWAS). We reported that CERENKOV has state-of-the-art performance (by two traditional measures and a novel GWAS-oriented measure, AVGRANK) in a comparison to nine other tools for identifying functional noncoding SNPs, using a comprehensive reference SNP set (OSU17, 15,331 SNPs). Given that SNPs are grouped within loci in the reference SNP set and given the importance of the data-space manifold geometry for machine-learning model selection, we hypothesized that within-locus inter-SNP distances would have class-based distributional biases that could be exploited to improve rSNP recognition accuracy. We thus defined an intralocus SNP “radius” as the average data-space distance from a SNP to the other intralocus neighbors, and explored radius likelihoods for five distance measures.

**Results:**

We expanded the set of reference SNPs to 39,083 (the OSU18 set) and extracted CERENKOV SNP feature data. We computed radius empirical likelihoods and likelihood densities for rSNPs and control SNPs, and found significant likelihood differences between rSNPs and control SNPs. We fit parametric models of likelihood distributions for five different distance measures to obtain ten log-likelihood features that we combined with the 248-dimensional CERENKOV feature matrix. On the OSU18 SNP set, we measured the classification accuracy of CERENKOV with and without the new distance-based features, and found that the addition of distance-based features significantly improves rSNP recognition performance as measured by AUPVR, AUROC, and AVGRANK. Along with feature data for the OSU18 set, the software code for extracting the base feature matrix, estimating ten distance-based likelihood ratio features, and scoring candidate causal SNPs, are released as open-source software CERENKOV2.

**Conclusions:**

Accounting for the locus-specific geometry of SNPs in data-space significantly improved the accuracy with which noncoding rSNPs can be computationally identified.

**Electronic supplementary material:**

The online version of this article (10.1186/s12859-019-2637-4) contains supplementary material, which is available to authorized users.

## Background

### The rSNP detection problem

Human genome-wide association studies (GWAS) have led to the discovery of genetic variant-to-trait associations in thousands of studies collectively involving millions of individuals [1]. Functional interpretation of genetic loci identified through GWAS has primarily focused on *coding regions* in which single nucleotide polymorphisms (SNPs) can be mapped to consequence predictions based on amino acid changes [2]; however, 90% of human GWAS-identified SNPs are located in *noncoding* regions [3]. Within a noncoding trait-associated region, it is difficult to pinpoint the regulatory SNP (or rSNP) that is causal for trait variation [4]. Various types of SNP annotations that correlate with functional rSNPs are known [5], for example, phylogenetic sequence conservation [6] and expression quantitative trait locus (expression QTL, or eQTL) association [7]. But the general problem of how to integrate various types of genomic, phylogenetic, epigenomic, transcription factor binding site (TFBS), and chromatin-structural rSNP correlates in order to identify rSNPs is a fundamental challenge in computational biology. Progress on this problem has been spurred by the growth of literature-curated databases of experimentally validated rSNPs such as the Human Gene Mutation Database [8] (HGMD), ORegAnno [9] or ClinVar [10]. While various approaches to the rSNP recognition problem have been proposed that do not involve training based on an example set of experimentally validated rSNPs (we call such methods “unsupervised” approaches) [11–21], converging lines of evidence from our work [22] and others’ [23–26] suggest (but are not *entirely* consistent on this point [21]) that approaches that are supervised by example sets of experimentally validated rSNPs significantly improves accuracy with which rSNPs can be discriminated from nonfunctional noncoding SNPs.

Many types of genomic data have been used to derive SNP annotation features that have proved useful in supervised models for rSNP recognition [22]. The picture emerging from dozens of studies over the past ten years is that increasing the breadth and diversity of such SNP annotation features improves rSNP detection, and thus there has been a steady increase in the number of features that are used in machine-learning approaches for this problem, from 23 features [23], to 28 features [27], to 158 features [28], to 175 features [24], to 246 features in our previous work [22]. The dimensionality of feature-spaces has rapidly increased in the last few years, with reports of rSNP recognition models that incorporate 919 features [16, 26, 29] derived from epigenomic data from the Encyclopedia of DNA Elements (ENCODE) project [30] and 2132 features [25] derived from the Gene Ontology [31]. However, in our previous work [22] we found that a model with a 246-dimensional feature space clearly outperformed models [25, 26, 29] with significantly higher-dimensional feature spaces. This suggests that feature-feature correlation within, and sparsity of, high-dimensional feature-sets may lead to diminishing returns in terms of improving rSNP detection accuracy.

A variety of supervised classification algorithms have been proposed for identifying functional noncoding SNPs, including the support vector machine (SVM) [17, 19, 23, 32], naïve Bayes [27], ensemble decision tree algorithms [24, 25, 28], probabilistic graphical models [18, 33], deep neural networks [20, 26, 29], weighted sum of feature ranks [34], and our work using regularized gradient boosted decision trees [22] and deep residual networks [35]. Recently, there have been several proposals of hybrid methods such as combining recurrent and convolutional neural networks [26] and integrating deep neural networks with regularized gradient boosted decision trees [29]. Beyond binary classification, regression-based approaches have been proposed for detecting rSNPs, including linear regression [36] and a mixture-of-regressions model [37]. Overall, there has been a shift toward models with higher parametric complexity as the sizes of example sets of experimentally validated rSNPs has increased [22].

### Novelty and performance of our previous CERENKOV method

In our previous work [22], we described CERENKOV (Computational Elucidation of the REgulatory NonKOding Variome), a machine-learning approach for rSNP recognition that incorporated four key innovations. First, CERENKOV incorporated a within-group-rank-based measure of classification accuracy, which we called AVGRANK. AVGRANK more realistically models the costs associated with incorrect predictions in post-GWAS SNP analysis than typical measures of accuracy like area under the receiver operating characteristic (AUROC) curve or area under the precision-vs-recall (AUPVR) curve. We found that optimizing a model to maximize AUPVR does not guarantee optimality for AVGRANK, and thus, that both measures should be taken into account in evaluating the performance of a computational model for rSNP recognition. Second, in CERENKOV we used a state-of-the-art regularized gradient boosted decision tree (xgboost) classification algorithm [38], which improved upon the rSNP recognition performance that could be achieved (on an identical feature-set) using the previously-proposed classification algorithms Random Forest and Kernel Support Vector Machine [22]. Third, for CERENKOV we engineered 246 SNP-level features from phylogenetic, genomic, epigenomic, chromatin structural, cistromic, population genetic, replication-timing, and functional genomic datasets. Fourth, we trained, validated, and performance-benchmarked CERENKOV using a reference set of 15,331 SNPs (the OSU17 SNP set) comprising 1659 experimentally validated human rSNPs and 13,672 neighboring “control” SNPs (cSNPs) that are each in strong linkage disequilibrium with at least one rSNP. We selected the OSU17 SNPs to represent noncoding loci that would be expected to be encountered in a post-GWAS analysis, based on population minor allele frequency [22]. We compared the accuracy of CERENKOV to nine other published rSNP recognition models (DeltaSVM [19], RSVP [25], DANN [20], fitCons [18], CADD [17], DeepSEA [29], DANQ [26], Eigen [21], and GWAVA [24]) and found that CERENKOV’s performance significantly improved upon the current state-of-the-art, by AUPVR, AUROC, and AVGRANK.

### Introducing CERENKOV2

In this work we report on CERENKOV2, a next-generation machine-learning approach for rSNP recognition that improves upon our previous approach, CERENKOV [22] in terms of accuracy and insights into the data-space geometry of the problem. In addition to using a significantly expanded reference set of SNPs [the OSU18 SNP set (see “The OSU18 reference SNP set” section), which has 39,083 SNPs for model benchmarking], we have incorporated new engineered features into CERENKOV2 that are based on likelihood ratios of average SNP-to-neighboring-SNPs distances for various types of distance measures, as described below. By taking account geometric properties of the distribution of SNPs in data space (as described in detail in the next section), CERENKOV2 achieves significantly better rSNP recognition performance than CERENKOV.

### The importance of data-space geometry

It is a well-established principle in machine-learning that understanding the manifold structure of cases in data-space can help guide appropriate selection of a classification model and/or geometric features that enable more accurate classification [39, 40]. Data-space inter-sample distance measures are fundamental to many machine-learning algorithms such as *k*-Nearest-Neighbors [41] (*k*-NN), and in the case of *k*-NN, the choice of distance measure can be a key determinant of the accuracy of the classifier [42]. Given that (1) rSNPs and cSNPs are grouped into genetic loci in which the within-locus SNPs are in linkage disequilibrium with one another (making rSNP recognition a *grouped* machine-learning problem), and (2) in the reference SNP set, each associated locus has at least one rSNP in it and usually many cSNPs (such that the problem has a “sparse positive bag” structure [43, 44]), we hypothesized that within-locus SNP-SNP distances in data space may be informative for discriminating rSNPs from cSNPs. But despite the importance of the choice of data-space metric in many machine-learning applications and in clustering [45], the potential utility of data-space metric-based features for improving accuracy of computational recognition of rSNPs has not to our knowledge been systematically explored. Here we report on the first effort (of which we are aware) to improve rSNP detection performance by systematically incorporating data-space geometric features, specifically, intralocus SNP-SNP distances in feature space.

### Data-space geometric features for rSNP recognition

Based on our initial observation that SNPs within the same locus tend to be clustered in data space, we investigated whether there are class label-specific biases in the locus-based average SNP-to-neighboring-SNPs distances that could be exploited to improve accuracy for discriminating rSNPs from cSNPs. In mathematical terms, for a SNP *s*, we denote by *L*(*s*) the set of SNPs within the same locus as *s* (for details on the selection of cSNPs that are within the same locus as an rSNP, see “Methods” section). Then, for a given locus *s* and a given distance metric *d*(·,·), we define an intralocus average SNP-to-neighboring-SNP distance or “intralocus radius” *λ*_*s*|*d*_ by

1 $$ \lambda_{s \vert d} = \frac{1}{\vert L(s) \vert - 1} \sum_{s^{\prime} \in L(s), s^{\prime} \neq s} d(s, s^{\prime}).  $$

**Fig. 1:**
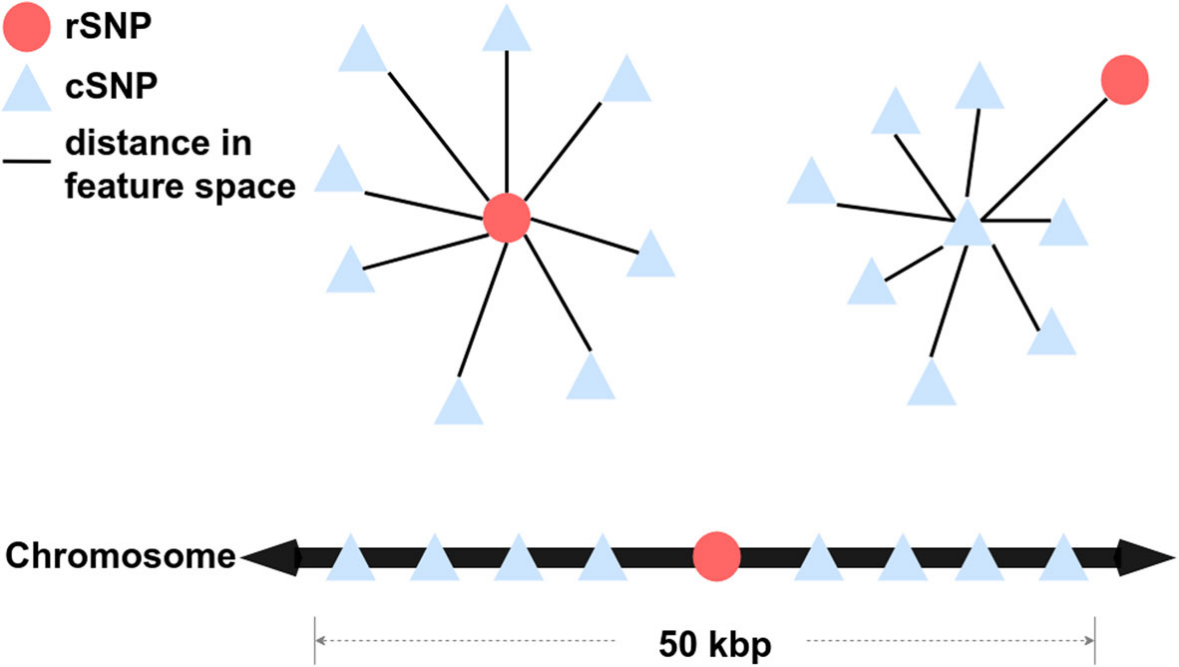
The geometric idea behind the intralocus distance features that are used in CERENKOV2. Top panel, SNPs from the same locus form a data-space “cloud.” Triangles and circles, SNPs; black lines, distances between a central SNP and the other SNPs within the locus. Bottom panel, SNPs shown in their chromosomal context

One such metric would be the Pearson distance defined as *d*(*s*,*s*^′^)=1−*r*(*s*,*s*^′^), where *r*(*s*,*s*^′^) is the Pearson correlation coefficient [46] between the feature vectors of SNPs *s* and *s*^′^. With Pearson distance being applied, we found that the distribution of intralocus radii for rSNPs were markedly different from cSNPs’, with rSNPs often having higher intralocus radii than cSNPs, i.e., *λ*_*r*|Pearson_>*λ*_*c*|Pearson_. Given the sparsity of rSNPs in the genome (cSNPs outnumber rSNPs 14.5 to one in the OSU18 SNP set) and the typically large linkage disequilibrium-defined locus sizes in the human genome [47], the locus neighborhood for any given *s* in general mostly contains cSNPs. Together, these observations suggest that in feature-space, the SNPs of a given locus have an “atom”-like structure with respect to Pearson distance–a core rSNP and a “cloud” of cSNPs with higher average distance from the it (Fig. 1).

Based on this initial observation, we systematically calculated intralocus radii for each SNP in the OSU18 reference SNP set, using five different distance measures (Canberra [48], Euclidean [49], Manhattan [50], cosine [51], and Pearson) applied to both scaled and unscaled feature data (for a total of ten combinations). We found significant differences between the distributions of the ten intralocus radius values conditioned on the two classes (rSNPs and cSNPs). Based on this, we parametrically modeled the intralocus radius distributions (see “Analysis of intralocus radius distributions for rSNPs and cSNPs” section) and thereby obtained log-likelihood ratios that we incorporated into the feature set for CERENKOV2 (see “Using data-space geometric features in CERENKOV2” section). We quantified the relative importance of the distance based features in the context of the CERENKOV2 base feature-set (see “CERENKOV2 feature importance” section). Finally, we compared the classification performance of CERENKOV2—including the new distance-based features—with that of CERENKOV on the OSU18 reference SNP set (see “Comparison of CERENKOV2 vs. CERENKOV performance” section) and found that CERENKOV2 had significantly better performance than CERENKOV, by AUROC, AUPVR, and AVGRANK. The complete feature data for the OSU18 training and validation SNP set are available online and the software code for CERENKOV2 is freely distributed to the scientific community online under an open-source license (see “Availability of data and materials” section).

## Results

### Analysis of intralocus radius distributions for rSNPs and cSNPs

**Fig. 2:**
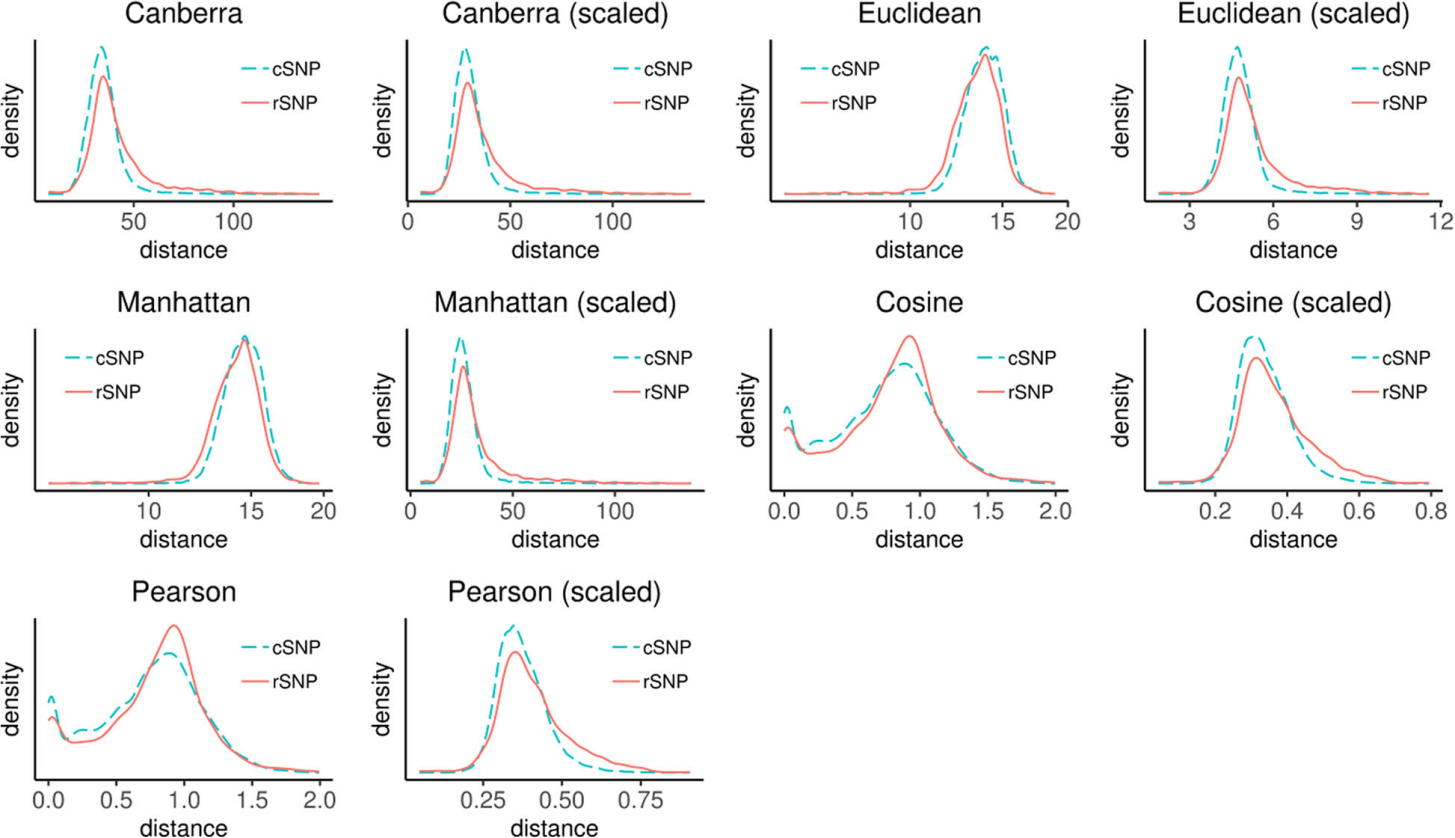
Distributions of intralocus radii computed using five different distance measures (Canberra, Euclidean, Manhattan, cosine, and Pearson) applied to scaled and unscaled feature data, conditioned on the type of reference SNP (rSNP or cSNP) for the intralocus radius calculation. Results shown are for all OSU18 SNPs (see “The OSU18 reference SNP set” section). Significant differences in the rSNP likelihoods vs. cSNP likelihoods are evident for Canberra, Canberra (scaled), Euclidean (scaled), Manhattan (scaled), cosine, and Pearson methods for computing intralocus radii. Modest differences in rSNP vs. cSNP likelihoods were evident for the cases of Euclidean and Manhattan methods for computing intralocus radii

We computed intralocus radii for each of the OSU18 SNPs (see “Computing the geometric features” section) using ten combinations of distance measures and data matrices: Canberra distance, Euclidean distance (*L*^2^ norm), Manhattan distance, cosine distance (defined as 1.0 minus the cosine similarity) and Pearson distance, each on unscaled data and min-max scaled data (the latter set of distance measures will be designated with the suffix “(scaled)” in each case). We first analyzed the intralocus SNP-SNP radius distributions for the two SNP classes (rSNPs and cSNPs) within 248-dimensional feature-space using kernel density estimation for radius values conditioned on the class label (rSNP or cSNP) of the reference SNP. As seen in Fig. 2 (see also Additional file 1: Table S2), there are class label-dependent differences in the skewness and kurtosis, indicting that geometric biases exist between rSNPs and cSNPs in data-space.

For cosine and Pearson distances, the intralocus radius distributions for rSNPs are slightly more skewed to the left and more platykurtic than the distributions for cSNPs; in terms of Euclidean and Manhattan distances, the intralocus radius distributions for rSNPs are left-skewed and more leptokurtic, while the cSNPs’ are right-skewed and less leptokurtic; for the rest distances, the intralocus radius distributions for cSNPs are slightly more skewed to the right and more leptokurtic than the distributions for rSNPs (see also Additional file 1: Table S2).

### Analysis of intralocus radius likelihood ratios (rSNP vs. cSNP)

**Fig. 3:**
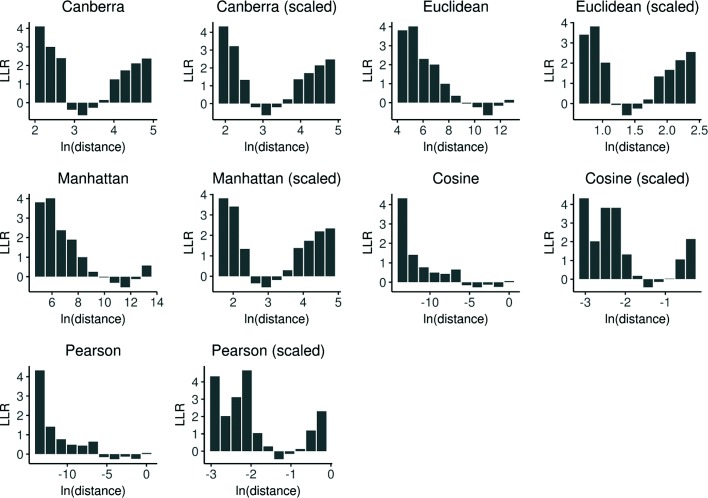
Empirically estimated log-likelihood ratios (rSNP/cSNP) based on intralocus radii computed using ten methods. Results shown are for all OSU18 SNPs (see “The OSU18 reference SNP set” section). LLR, log-likelihood ratio (natural logarithm); ln, natural logarithm

The intralocus radius distribution analysis suggested that taking account of the intralocus radius likelihood for the SNP conditioned on a possible class label (rSNP or cSNP) would be useful for discriminating rSNPs from cSNPs. To visualize the potential class-label discriminating power of each of the ten methods for computing intralocus radii, we empirically estimated the rSNP/cSNP log-likelihood ratios (LLRs) for the ten different methods for computing intralocus radii using binned counts of SNPs for posterior probability estimation (Fig. 3). Consistent with the differences seen in the density distributions (Fig. 2), we found that log-likelihood ratios were significantly different from zero for the majority of bins for intralocus radii computed, for each of the ten distance measures except for cosine (unscaled) and Pearson (unscaled).

Next, we extracted features from intralocus radii for use in the CERENKOV classifier, using sets of SNPs that were reserved for training within a cross-validation framework (see “Gradient boosted decision trees” section). In order to avoid issues with zero-count bins associated with the limited number of SNP loci within a single cross-validation fold, we used a parametric approach: instead of empirically estimating likelihood ratios, for each of the ten methods for computing intralocus radii we fit parametric distributions to the radius values (conditioned on the class label of the reference training SNP). We then applied the fitted parametric models to compute log-likelihood ratios for both the training and validation sets of SNPs and integrated those ten log-likelihood ratios as feature vectors, yielding a 258-column feature matrix input for classification which we compared to performance (using the same classification algorithm) of the original 248-column feature matrix.

### Using data-space geometric features in CERENKOV2

On an identical starting set of reference SNPs (OSU18, see “The OSU18 reference SNP set” section) and identical assignments of SNPs to cross-validation folds, we compared the performance of the CERENKOV classification algorithm incorporating the 248-column feature matrix (without intralocus radii-based features) with the performance of the CERENKOV algorithm incorporating a 258-column feature matrix (including intralocus radii-based features). Using ten independent replications of five-fold cross-validation with grouped sampling based on locus (“locus-based sampling”, see “Gradient boosted decision trees” section) and using three metrics (AUPVR, AUROC, and AVGRANK [22]), we measured performance separately for classification using the two feature matrices and using xgboost hyperparameters selected to maximize training-set AUPVR (see “Hyperparameter tuning” section). For the classification algorithm we used a high-performance implementation of regularized gradient boosted decision trees (xgboost [38], hereafter, xgboost-GBDT). For the two models, the inputs to xgboost were thus a 39,083 ×248 feature matrix and a 39,083 ×258 feature matrix, respectively. We trained and tested xgboost-GBDT (using ten independent replications of five-fold [52] cross-validation with locus-based sampling [22]) with the optimal xgboost hyperparameters (see “Hyperparameter tuning” section).

### Comparison of CERENKOV2 vs. CERENKOV performance

**Fig. 4:**
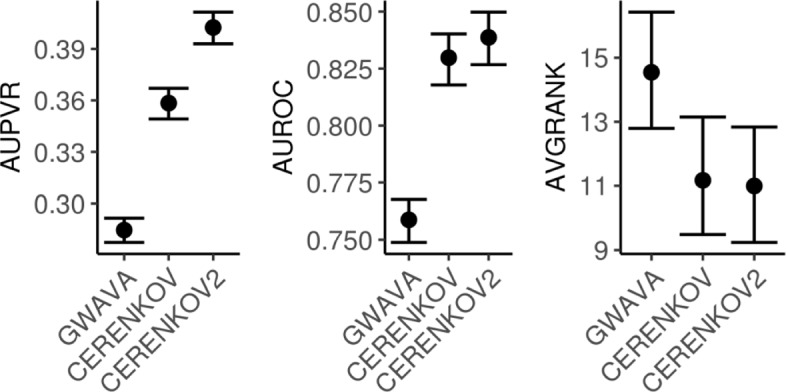
Performance of GWAVA, CERENKOV and CERENKOV2 on the OSU18 reference SNP set, by three performance measures. Marks, sample arithmetic mean of validation-set performance; bars, estimated 95% confidence intervals (see “Gradient boosted decision trees” section); GWAVA, based on the GWAVA’s Random Forest model with 174 features [24]; CERENKOV, our previous model with the base 248-column feature matrix; CERENKOV2, our current model consisting of the base feature matrix plus ten log-likelihood features derived from intralocus radii and fitted using training data only; AUPVR, area under the precision-vs-recall curve (higher is better); AUROC, area under the receiver operating characteristic curve (higher is better); AVGRANK, intralocus average score rank (lower is better [22])

Within the above-described cross-validation framework, we found that the inclusion of the ten geometric features improved validation-set AUPVR from 0.358 to 0.402 (*p*<10^−25^), AUROC from 0.830 to 0.839 (*p*<10^−18^), and AVGRANK from 11.172 to 10.994 (lower is better for AVGRANK [22]; *p*<0.004) (Fig. 4 and Additional file 1: Table S1). From these results, we concluded that the addition of the ten geometric features based on the intralocus radius of SNPs in data-space significantly improved performance for rSNP recognition.

### CERENKOV2 feature importance

**Fig. 5:**
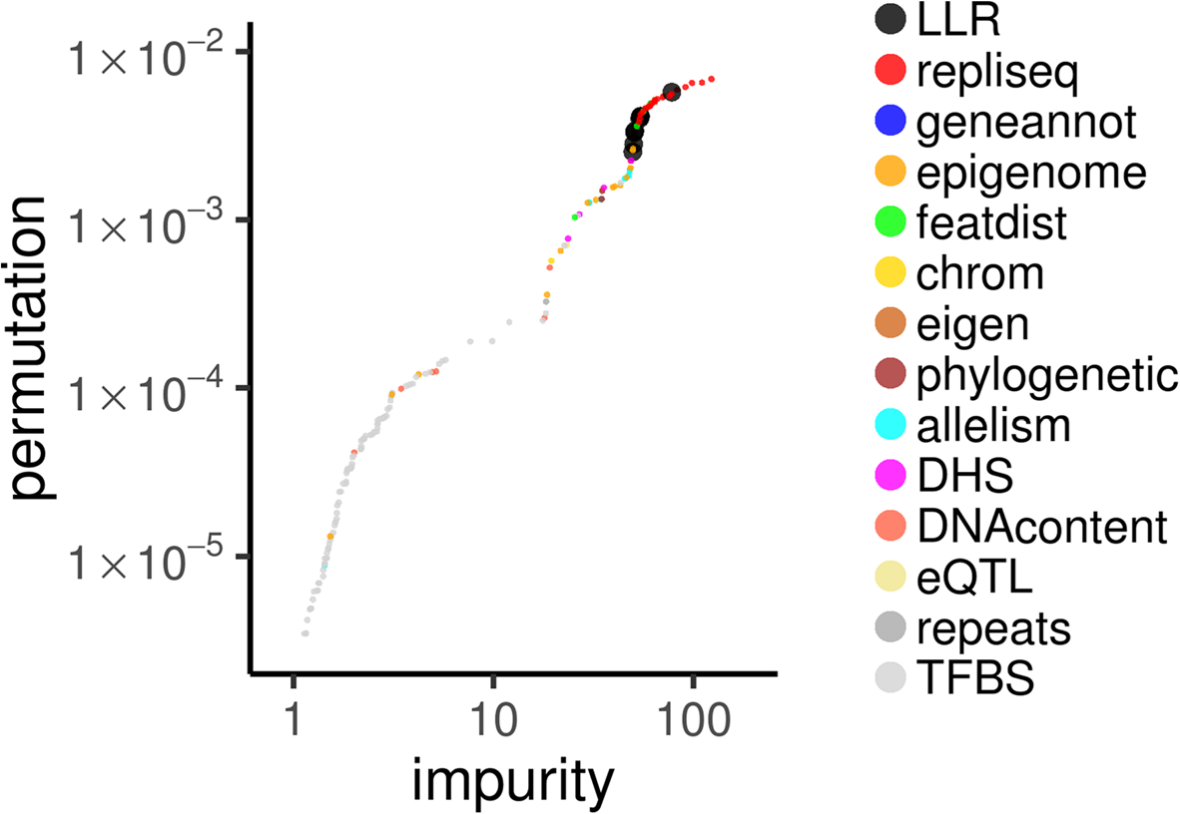
Gini and permutation importance values of 258 features in 14 categories (colored marks). Feature category labels as follows: “LLR”, log-likelihood ratio (the new data-space geometric features); “repliseq”, replication timing; “geneannot”, gene-model annotation-based; “epigenome”, epigenomic segmentation [67, 68]; “featdist”, SNP location-related; “chrom”, the chromosome; “eigen”, based on the Eigen [21] score; “phylogenetic”, phylogenetic interspecies local sequence conservation [6, 80, 81]; “allelism”, allele and MAF-related; “DHS”, DNase I hypersensitive site; “DNAContent”, local nucleotide frequences; “eQTL”, expression quantitative trait locus [75]; “repeats”, genomic repeat annotation; “TFBS”, transcription factor binding site (see “Extracting the nongeometric features” section and Ref. [22] for details)

In order to better understand the contributions of different categories of features—particularly geometric features—to rSNP recognition accuracy, we separately trained a Random Forest algorithm on the 258-column feature matrix for the OSU18 reference SNP set (see “The OSU18 reference SNP set” section) and then obtained permutation [53] and Gini impurity [54]-based estimates of the importance of each of the 258 features (Fig. 5). Consistent with findings from the Peterson et al. study [25], SNP annotations based on replication timing experimental measurements (“repliseq”) had highest overall feature importance; however, the ten log-likelihood-ratio features that were based on data-space geometry strongly contributed to accuracy for rSNP recognition.

### Application of CERENKOV2 to identify trait-associated noncoding SNPs

To illustrate the biological utility of CERENKOV2, we used CERENKOV2 to compute rSNP prediction scores for noncoding SNPs in the Genome-Wide Repository of Associations Between SNPs and Phenotypes (GRASP) database. We identified two noncoding SNPs that are trait-associated in GRASP and that have CERENKOV2 rSNP prediction scores greater than 0.7: rs2239633 (associated with acute lymphoblastic leukemia), and rs11071720 (associated with mean platelet volume in circulation, and with gene expression of *TPM1* in blood. This illustrates how CERENKOV can be used to filter GWAS summary results to identify noncoding SNPs that have high potential to have a mechanistic (gene regulatory) interpretation.

## Discussion

We anticipate that CERENKOV2’s performance may be improved through several possible enhancements. An appealing extension would be to combine deep neural network-based approaches based on the local 1 kbp sequence haplotype (recognizing that the local haplotype provides important correlates of functional SNP alleles [55]), with CERENKOV2’s current set of 258 SNP features. Our previous work [35] has demonstrated that a classifier (Res2s2am) based on a deep residual network architecture has state-of-the-art performance on the related problem of discriminating trait-associated noncoding SNPs from control noncoding SNPs. While the present work significantly improves rSNP recognition accuracy, the validation-set AVGRANK performance values (averaging nearly 11) clearly demonstrate that further gains in accuracy are needed in order to fully realize the potential of integrative, data-driven computational approaches to substantially accelerate the search for causal noncoding GWAS variants. Undoubtedly, precision values are dampened by “latent positives” in the training dataset, i.e., high-scoring cSNPs that are simply undiscovered rSNPs. Using machine learning techniques that are specifically designed to address “positives-plus-unlabeled” problems [56] (such as the rSNP detection problem studied here) or semi-supervised learning algorithms [57] would seem to offer a principled approach to handling the issue of latent positives among the cSNPs. Given the extent to which common features (e.g., replication timing, local GC content, phylogenetic sequence conservation, chromatin accessibility, and transcription factor binding sites [22]) are used by many supervised tools for rSNP recognition, the results from our analysis of the performance of CERENKOV2 suggest that accounting for the intralocus data-space geometry of SNPs may be broadly useful for advancing bioinformatics for post-GWAS SNP analysis.

## Conclusion

CERENKOV2 significantly improves upon our previous framework and classifier, CERENKOV, in its ablity to score noncoding SNPs based on their regulatory potential. CERENKOV2—by virtue of its training-set construction criteria (locus-based, MAF ≥ 0.05) and its novel feature set including geometric ones—is specifically designed for the problem of identifying candidate causal noncoding SNPs in GWAS summary regions. We have demonstrated, using side-by-side comparisons on identical assignments of SNPs to cross-validation folds, that CERENKOV2’s performance exceeds that of our previous CERENKOV, by both classical global rank-based measures (AUPVR and AUROC) and by the GWAS-oriented performance measure (AVGRANK) that we previously proposed. In particular, CERENKOV2’s validation-set AUPVR performance, 0.404, is a significant improvement over CERENKOV’s AUPVR of 0.359 on the same reference SNP set (OSU18). The results reported in this work are based on a significantly expanded reference SNP set (OSU18, which has more than double the number of SNPs in the OSU17 reference set), which should increase the generalizability and robustness of the performance results reported herein.

The source code, feature data files, and instructions for installing and running CERENKOV2 are freely available online (see “Availability of data and materials” section). By making the software, the data files, and in particular the OSU18 SNP set (with benchmark results) available, we hope to accelerate development of methods for functional analysis of noncoding SNPs and ultimately increase the yield of molecular insights from GWAS.

## Methods

### The OSU18 reference SNP set

We obtained minor allele frequencies (MAFs) for all SNPs from the dbSNP-based [58] snp146 SQL table hosted at the UCSC Genome Browser [59] site. For the representative set of rSNPs for training/evaluation, we obtained 2,529 rSNPs in total from HGMD (Rel. 2017.2), ORegAnno (Rel. 2015.12.22) and ClinVar that satisfied all of the following criteria: (i) for all SNPs from HGMD, they were marked as regulatory in HGMD and the disease field did not contain cancer; the other SNPs from ORegAnno or ClinVar were of GRCh37 (hg19) assembly; (ii) MAF≥0.05; (iii) the SNP was not an indel and not contained within a coding DNA sequence (CDS; based on the complete set of transcripts from the Ensembl 75 gene annotation build); and (iv) the SNP was not exclusively mapped to the Y chromosome (due to the lack of phased haplotype data available for proxy SNP searching). For each of these rSNPs, we used the SNP Annotation and Proxy Search (SNAP) tool [60] to identify SNPs that are in LD (*r*^2^≥0.8 in 1,000 Genomes (1KG) Phase 1 [61], with data from the International HapMap Project [62] used instead of 1KG for chromosome X), and we filtered to include only SNPs within 50 kbp of an rSNP, that were not contained within a CDS, that have MAF≥0.05, and that are not themselves on the list of rSNPs. Overall, this filtering procedure produced a list of 36,554 cSNPs. The combined set of 39,083 SNPs (which we call the OSU18 reference SNP set) was thus designed as an appropriate reference set for the application of post-GWAS SNP analysis. Overall, the class imbalance of OSU18 is ∼14.454 (cSNP/rSNP).

### Extracting the nongeometric features

The CERENKOV feature extraction software is based on Python and SQL. We extracted 248 SNP features for each of the OSU18 SNPs, using information and measurements from SNP annotation databases, epigenomic and chromatin datasets and phylogenetic conservation scores (Table 1).

**Table 1:** The 248 SNP features used in CERENKOV

*Feature(s)*	*Feature type*	*Raw data src.*	*Feature description*
normChromCoord	continuous	UCSC	the SNP coordinate (normalized to chrom. length)
majorAlleleFreq	continuous	UCSC/1KG	the major allele frequency (1KG)
minorAlleleFreq	continuous	UCSC/1KG	the next-to-major allele frequency (1KG)
phastCons	continuous	UCSC	46-way placental mammal phastCons score [6]
GERP ++	continuous	UCSC	bp-level GERP ++ [80] score
avg_GERP	continuous	UCSC	avg. GERP score [81] in ±100 bp window
avg_daf	continuous	1KG	average derived allele frequency in ±1 kbp region
avg_het	continuous	1KG	average heterozygosity rate in ±1 kbp region
maf1kb	continuous	UCSC/1KG	average of the MAF values for all SNPs in ±1 kbp window
eqtlPvalue	continuous	GTEx	-log_10_ min(*p*) for GTEx eQTL for the SNP, across 13 tissues [75]
GC5Content	integer (0-5)	UCSC	GC content in a 5 bp window
GC7Content	integer (0-7)	UCSC	GC content in a 7 bp window
GC11Content	integer (0-11)	UCSC	GC content in a 11 bp window
local_purine	integer (0-11)	UCSC	number of purine bases in local 11 bp window
local_CpG	integer (0-10)	UCSC	number of CpG dinucleotides in 11 bp window
ss_dist	integer	UCSC	signed distance to nearest exon boundary
tssDistance	integer	Ensembl75	signed distance to nearest Ensembl TSS
gencode_tss	integer	GENCODE	signed distance to nearest GENCODE TSS
tfCount	integer	UCSC	sqrt(count) of ENCODE ChIP-seq TFBS overlap. SNP
uniformDhsScore	integer	UCSC	sum scores of ENCODE uniform DHS peaks overlap. SNP
uniformDhsCount	integer	UCSC	count of ENCODE uniform DHS peaks overlap. SNP
masterDhsScore	integer	UCSC	sum scores of ENCODE master DHS peaks overlap. SNP
masterDhsCount	integer	UCSC	count of ENCODE master DHS peaks overlap. SNP
chrom	categorical (23)	UCSC	the chromosome to which the SNP maps
nestedrepeat	categorical (2)	UCSC	SNP is in a RepeatMasker [70] DNA repeat
simplerepeat	categorical (2)	UCSC	SNP is in a Tandem Repeats Finder [71] repeat
cpg_island	categorical (2)	UCSC	SNP is in an epigenome-predicted CpG island [72]
geneannot	categorical (4)	UCSC	classifies SNP location as CDS, intergenic, UTR, or intron
majorAllele	categorical (4)	UCSC/1KG	the major allele for the SNP
minorAllele	categorical (4)	UCSC/1KG	the next-to-major allele for the SNP
pwm	categorical (22)	Ensembl75	ID of the Jaspar 2014 [74] motif in which SNP is a match
chromhmm	6 ×categ. (26)	UCSC	ChromHMM label in Gm12878, H1hesc, HeLaS3, HepG2, HUVEC and K562 cells
segway	6 ×categ. (26)	UCSC	Segway label in Gm12878, H1hesc, HeLaS3, HepG2, HUVEC and K562 cells
ch_comb_WEAKENH	categorical (4)	Ensembl75	ChromHMM label in Ensembl Reg. Seg. build
ch_comb_ENH	categorical (6)	Ensembl75	ChromHMM label in Ensembl Reg. Seg. build
ch_comb_REP	categorical (7)	Ensembl75	ChromHMM label in Ensembl Reg. Seg. build
ch_comb_TSSFLANK	categorical (5)	Ensembl75	ChromHMM label in Ensembl Reg. Seg. build
ch_comb_TRAN	categorical (7)	Ensembl75	ChromHMM label in Ensembl Reg. Seg. build
ch_comb_TSS	categorical (7)	Ensembl75	ChromHMM label in Ensembl Reg. Seg. build
ch_comb_CTCFREG	categorical (7)	Ensembl75	ChromHMM label in Ensembl Reg. Seg. build
ENCODE_TFBS	160 ×categ. (2)	UCSC	160 features for SNP being in an ENCODE TFBS [84] peak
FsuRepliSeq	16 ×continuous	UCSC	Replication Timing by Repli-chip [66] from ENCODE/FSU
UwRepliSeq	16 ×continuous	UCSC	Replication Timing by Repli-seq [65] from ENCODE/UW
SangerTfbsSummary50kb	continuous	Ensembl75	Summary of Ensembl TFBS peaks from 18 human cell types
NkiLad	categorical (2)	UCSC	SNP is in a Lamina Associated Domain (NKI study [85], Tig-3 cells)
vistaEnhancerCnt	categorical (2)	UCSC	count of VISTA [73] HMR-Conserved Non-coding Human Enhancers [86] overlap. SNP
vistaEnhancerTotalScore	categorical (2)	UCSC	sum scores of VISTA [73] HMR-Conserved Non-coding Human Enhancers [86]
eigen	continuous (2)	Eigen	Eigen & Eigen-PC v1.1 raw scorea [21]

Abbreviations are as follows: UCSC, UC Santa Cruz Genome Browser portal; 1KG, 1,000 Genomes Project; Ensembl75, Ensembl Release 75 [82]; GENCODE, the GENCODE project release 19 [83]; ENCODE, Encyclopedia of DNA Elements [30]; FSU, Florida State University; UW, University of Washington; NKI, Netherlands Cancer Institute; GTEx, the genotype tissue-expression project; GERP, the Genomic Evolutionary Rate Profiling score; CDS, coding DNA sequence; UTR, untranslated region; MAF, minor allele frequency; HMR, human-mouse-rat; TSS, transcription start site

#### Features extracted from UCSC

We used the snp146 UCSC SQL table as the initial source for SNP annotations (GRCh37 assembly coordinate system). We extracted additional SNP annotation information by (i) coordinate-based joins to other genome annotation tracks in the UCSC database; and (ii) by joining with non-UCSC data sources using the SNP coordinate. For triallelic and quadrallelic SNPs, we used the two most frequent alleles, for the purpose of obtaining features that depend on allele-dependent scores. We derived DNase I hypersensitive site (DHS) features from data tracks from published genome-wide assays with high-throughput sequencing-based detection (DNase-seq) from the ENCODE project [63] (the master peaks are summary peaks combining data from DHS experiments in 125 cell types; the uniform DHS peaks are from DHS experiments in individual cells, processed using the ENCODE uniform peaks analysis pipeline [64]). The ENCODE_TFBS feature is presented in Table 1 as a single feature for conciseness, but in fact it is 160 separate binary features, one for each transcription factor (TF) for which genome-wide TFBS data (from chromatin immunoprecipitation with high-throughput sequencing readout, or ChIP-seq) and peak data (from the ENCODE Uniform Peaks analysis) are available [64]. For replication timing features, we processed track-specific BigWig files for Repli-seq [65] and Repli-chip [66] experiments from UCSC to obtain the timing scores at individual SNP positions. For ChromHMM [67], Segway [68] and lamina-associated domains (LAD) [69] annotations, we used the SQL tables from UCSC. We used BED file downloads to obtain annotations for DNA repeat elements predicted by RepeatMasker [70], DNA repeat elements predicted by Tandem Repeats Finder [71], epigenome-based CpG island predictions produced by the Bock et al. software pipeline [72], and VISTA enhancer predictions [73].

#### Features extracted from Ensembl

We used the BioMart tool to download (i) TFBS motif occurrences (based on the 2014 release of the Jaspar database [74]) and ChromHMM chromatin segmentation labels from Ensembl Regulation 75 and (ii) GENCODE transcription start sites (TSS; from Ensembl Genes 75) with which we computed signed TSS distances.

#### GTEx feature

We obtained SNP-to-gene associations for 13 tissues (adipose, artery/aorta, artery/tibial, esophagus/mucosa, esophagus/muscularis, heart left ventricle, lung, skeletal muscle, tibial nerve, sun-exposed skin, stomach, thyroid, and whole blood) from the Genotype Tissue Expression (GTEx) Project [75] Analysis Version 4 from the GTEx project data portal. For each SNP, we selected the minimum association *p*-value across genes and tissues.

### Computing the geometric features

For each distance metric *d*(·,·), we first computed the intralocus radius *λ*_*s*|*d*_ for each SNP *s* in our OSU18 dataset, in the data-space of all the 248 features (categorical data were binary-encoded which expanded the dimension of the data space to 587); then we separated those intralocus radii according to SNP classes, making two sets *Λ*_*r*|*d*_={*λ*_*r*|*d*_|*r* is an rSNP} and *Λ*_*c*|*d*_={*λ*_*c*|*d*_|*c* is a cSNP}. For empirical estimation of likelihoods, we used the R hist function with 11 bins on *Λ*_*r*|*d*_ and *Λ*_*c*|*d*_ and then gathered the bin counts, $\left \lbrace N_{r \vert d}^{(1)}, \dots, N_{r \vert d}^{(11)}\right \rbrace $ and $\left \lbrace N_{c \vert d}^{(1)}, \dots, N_{c \vert d}^{(11)}\right \rbrace $ respectively. The empirical likelihood ratio for bin *i* can be computed with formula ${LR}_{d}(i) = \frac {N_{r \vert d}^{(i)}}{N_{c \vert d}^{(i)}}$. For fitting parametric density distributions for intralocus radii, we used the fitdistrplus package (version 1.0.9) [76] in R and we used the normal distribution for cosine and Pearson distances and the log-normal distribution for the other eight combinations of distance function and data scaling/non-scaling. Akaike information criterion was leveraged (AIC) [77] for distribution selection. Then for each distance metric *d*(·,·), 2 probability density functions, *p*_*r*|*d*_(·) and *p*_*c*|*d*_(·), can be estimated from *Λ*_*r*|*d*_ and *Λ*_*c*|*d*_. And for any given SNP *s*, its likelihood ratio is defined as the ratio of its probability densities, i.e. ${LR}_{d}(s) = \frac {p_{r \vert d}(\lambda _{s \vert d})}{p_{c \vert d}(\lambda _{s \vert d})}$. This likelihood can be interpreted as the extent to which SNP *s* inclines to be an rSNP, observing its intralocus radius.

For loci where only one SNP (rSNP in all cases) was located, we set its likelihood ratio to 1. For each of the OSU18 SNPs, and using the parametric distributions fitted as described above, we computed log-likelihood-ratio scores for each of the ten combinations of distance metric and scaled/unscaled data listed in “Data-space geometric features for rSNP recognition” section. [The rationale for using min-max scaling for the data matrix for Canberra, Euclidean, and Manhattan distances was to reduce the impact of high-variance continuous features]. The ten columns of log-likelihood-ratio data were then appended to OSU18 dataset as ten new features during our machine learning processes.

### Machine learning

The feature extraction and distance computation were done in Python 3 under Ubuntu 16.04 and would take about two hours with a single core of an Intel Core i7-4790 CPU. Peak RAM usage was approximately 12 GB.

For the machine learning framework, we used the R statistical computing environment (version 3.4.4) [78], also under Ubuntu 16.04. The complete machine-learning process required 25 min in total for the three models (GWAVA, CERENKOV and CERENKOV2).

#### Random forest

In order to compare CERENKOV2 with GWAVA [24], we annotated OSU18 dataset with the GWAVA program and then applied Random Forest algorithm to the gained GWAVA feature matrix. Specifically, we used the R package ranger [79] version 0.6.0 with the published hyperparameters. To make a fair comparison, we adapted the same cross-valiation settings and performance measurements to CERENKOV2’s (see “Gradient boosted decision trees” section below). In addition, Random Forest is also applied to illustrate CERENKOV2 feature importances (see “CERENKOV2 feature importance” section).

#### Gradient boosted decision trees

For the gradient boosted decision trees (GBDT) classifier, we used the R API for xgboost [38] version 0.6.4.1. We used gradient boosted trees (booster=gbtree) and binary logistic classification as the objective, with the default loss function (objective=binary:logistic). We used ten-fold cross-validation [52] with *locus-based sampling*, in which we assigned rSNPs to folds (stratifying on the number of cSNPs per rSNP), and then assigned cSNPs to the *same fold to which it’s LD-linked rSNP was assigned*. Thus, in the case of locus-based sampling, an rSNP and its linked cSNPs are always assigned to the same cross-validation fold. Especially for those 10 geometric features, distribution parameters were estimated only on training data to prevent data leakage. For every prediction performance metric we report, the fold composition was exactly the same across all of the rSNP recognition models studied. We set base_score = 0.06918531 (the rSNP/cSNP class imbalance). We estimated 95% confidence intervals on the sample mean using 1,000 iterations of bootstrap resampling [52].

#### Hyperparameter tuning

We tuned the xgboost-GBDT classifier with a hyperparameter septuple grid size of 3,888, with locus-based sampling. The tuning hyperparameter tuple that maximized the validation AUPVR was: *η*=0.1, *γ*=10, nrounds = 30, max_depth = 7, subsample = 1.0 and scale_pos_weight = 1; we used these hyperparameter values for all subsequent analyses using xgboost-GBDT. (In contrast, the hyperparameter tuple that minimized the validation AVGRANK was: *η*=0.1, *γ*=100, nrounds = 30, max_depth = 6, subsample = 0.85, colsample_bytree = 0.85, and scale_pos_weight = 8).

#### GRASP database

We downloaded the full GRASP 2.0.0.0 catalog in tab-delimited value (TSV) format and joined the GRASP data with the CERENKOV2 prediction matrix using the dbSNP refSNP ID as the join key. We then filtered the resulting data matrix to include only SNPs whose GRASP trait-association *P*-values were less than the accepted human genome-wide significance level (5 ×10^−8^) and whose CERENKOV rSNP prediction score was at least 0.7.

## Additional file

Additional file 1 Supplementary Tables. This PDF file contains 2 supplementary tables. The first one provides a view of comparison of validation-set performance measures between GWAVA, CERENKOV and CERENKOV2 on the OSU18 reference SNP set. The second one lists the skewnesses and kurtoses of intralocus radii computed using Canberra, Euclidean, Manhattan, cosine, and Pearson distances, applied to scaled and unscaled feature data, and conditioned on the type of reference SNP (rSNP or cSNP). (PDF 90 kb)

## Abbreviations

AIC: Akaike information criterion
AUPVR: Area under precision-vs-recall curve
AUROC: Area under receiver operating characteristic curve
AVGRANK: Average ranks of the prediction scores for all ground-truth rSNPs within their loci
CERENKOV: Computational Elucidation of the REgulatory NonKOding Variome
CDS: Coding sequence
cSNP: Control SNP
DHS: DNase I hypersensitive site
ENCODE: Encyclopedia of DNA elements
GBDT: Gradient boosted decision tree
GERP: Genomic evolutionary rate profiling
GTEx: Genotype tissue-expression project
GWAS: Genome-wide association study
HGMD: Human gene mutation database
HMR: Human-mouse-rat
*k*-NN: *k*-nearest-neighbors
LAD: Lamina-associated domain
LLR: Log-likelihood ratio
MAF: Minor allele frequency
NKI: Netherlands cancer institute
QTL: Quantitative trait locus
rSNP: Regulatory SNP
SNAP: SNP annotation and proxy search
SNP: Single nucleotide polymorphisms
SVM: Support vector machine
TF: Transcription factor
TFBS: Transcription factor binding site
