# Supplementary material for: CERENKOV2: improved detection of functional noncoding SNPs using data-space geometric features
Source: BMC Bioinformatics. 2019 Feb 6;20:63. doi: 10.1186/s12859-019-2637-4 (PMC6364436; doi:10.1186/s12859-019-2637-4)
Supplement: Supplementary file 1 — Supplementary Tables. This PDF file contains 2 supplementary tables. The first one provides a view of comparison of validation-set performance measures between GWAVA, CERENKOV and CERENKOV2 on the OSU18 reference SNP set. The second one lists the skewnesses and kurtoses of intralocus radii computed using Canberra, Euclidean, Manhattan, cosine, and Pearson distances, applied to scaled and unscaled feature data, and conditioned on the type of reference SNP (rSNP or cSNP). (PDF 90 kb) [file 12859_2019_2637_MOESM1_ESM.pdf]

Supplementary Table 1 Comparison of validation-set performance measures between GWAVA, CERENKOV and CERENKOV2 on the `OSU18` reference SNP set. AUPVR, area under the precision-vs-recall curve; AUROC, area under the receiver operating characteristic curve; AVGRANK, average intralocus score rank (lower is better [1]); GWAVA means the published Random Forest model with 174 features [2]; CERENKOV means the 248-column base feature matrix; CERENKOV2 means the 248-column base feature matrix with the addition of the ten geometric features; p-values were computed using Welch's t-test against CERENKOV2 performance. The geometric features are intralocus radius log-likelihood ratios computed using parametric models fitted using feature data only from SNPs in the *training* set.

Measure	GWAVA	CERENKOV	CERENKOV2
AUPVR	0.285 ($p = 8.4 \times 10^{-39}$)	0.358 ($p = 9.9 \times 10^{-26}$)	0.402
AUROC	0.759 ($p = 2.1 \times 10^{-35}$)	0.830 ($p = 2.6 \times 10^{-18}$)	0.839
AVGRANK	14.546 ($p = 8.6 \times 10^{-32}$)	11.172 ($p = 0.0035$)	10.994

References

1. Yao, Y., Liu, Z., Singh, S., Wei, Q., Ramsey, S.A.: Cerenkov: Computational elucidation of the regulatory noncoding variome. In: Proceedings of the 8th ACM International Conference on Bioinformatics, Computational Biology,and Health Informatics, pp. 79–88. ACM, Corvallis, OR (2017)
2. Ritchie, G.R.S., Dunham, I., Zeggini, E., Flicek, P.: Functional annotation of noncoding sequence variants. Nature Methods **11**(3), 294–296 (2014)

Supplementary Table 2 Skewnesses and kurtoses of intralocus radii computed using five different distance measures (Canberra, Euclidean, Manhattan, cosine, and Pearson) applied to scaled and unscaled feature data, conditioned on the type of reference SNP (rSNP or cSNP). Scaled feature data are generated from applying min-max scaling to the unscaled; Pearson distance is defined as 1.0 minus the Pearson correlation coefficient; cosine distance is defined as 1.0 minus the cosine similarity.

Distance	Class	Skewness	Kurtosis
Canberra	cSNP	2.47	14.23
	rSNP	2.09	6.19
Canberra (scaled)	cSNP	2.84	16.76
	rSNP	2.20	6.49
Euclidean	cSNP	0.01	0.37
	rSNP	-0.70	2.87
Canberra (scaled)	cSNP	2.06	10.31
	rSNP	1.66	3.97
Manhattan	cSNP	0.02	0.26
	rSNP	-0.71	2.79
Manhattan (scaled)	cSNP	3.39	22.65
	rSNP	2.47	7.85
cosine	cSNP	-0.21	-0.49
	rSNP	-0.30	-0.03
cosine (scaled)	cSNP	0.79	1.26
	rSNP	0.69	1.04
Pearson	cSNP	-0.21	-0.49
	rSNP	-0.30	-0.03
Pearson (scaled)	cSNP	0.87	1.72
	rSNP	0.79	1.24
